# An animal model study of osteochondral defect repair by human adipose stem cells and pomegranate fruit hydroalchoholic extract

**DOI:** 10.22038/AJP.2022.21243

**Published:** 2023

**Authors:** Ahmad Teimourinejad, Batool Hashemibeni, Hossein Salehi, Fateme Sadat Mostafavi, Mohammad Kazemi, Hamid Bahramian

**Affiliations:** 1 *Department of Anatomical Sciences, Medical School, Isfahan University of Medical Sciences, Isfahan, Iran*; 2 *Department of Genetic and Molecular Biology, Medical School, Isfahan University of Medical Sciences, Isfahan, Iran*

**Keywords:** Pomegranate, Human adipose stem cells, Fibrin scaffold, Chondrogenic repair

## Abstract

**Objective:**

Articular cartilage damages do not repair spontaneously. Tissue engineering is a promising approach to repair cartilage damage. Transforming growth factor-beta (TGF-β) members are the known induction factors in chondrogenic differentiation. However, hypertrophy of the chondrocytes resulting from mesenchymal stem cells (MSCs) induction by TGF-β is inevitable. Pomegranate fruit contains many ingredients which are useful in ensuring the health of organs. This study was designed to investigate the Pomegranate Fruit hydroalchoholic Extract (PFE) capability in human adipose derived stem cells (hASCs) differentiation into the chondrocytes on fibrin scaffold.

**Materials and Methods:**

Pomegranate fruit hydroalchoholic extract (PFE) was prepared. hASCs were isolated, expanded, labeled, and seeded on the fibrin scaffold. The constructs were divided into three groups including TGF-β3, PFE, and control. The constructs were induced for 14 days, then, the MTT assay, Real-Time Polymerase Chain Reaction (PCR), and histochemistry assessments were run, and finally, the constructs were transplanted into the knee defect of rats. The gross and histological assessments of the transplants were done after 8 weeks.

**Results:**

The viability rate, *COL2A1, Aggrecan (ACAN) *and *COL10A1* genes expression levels, and histological criterion of the PFE samples were significantly higher than that of the control. The macroscopic grades and histological results of the PFE samples were close to that of the TGF-β3. The number of positive cells for COLІI protein were higher significantly in the PFE group than the control.

**Conclusion:**

PFE was effective in the chondrogenic induction of hASCs. Further studies are needed to find out the events of the chondrogenic induction using PFE.

## Introduction

Hyaline cartilage is an avascular and aneural tissue that becomes defected due to aging or sports injuries and is not self-cured (Risbud and Sittinger, 2002; Steinert et al., 2007). Condensation of MSCs as an initial stage of cartilage tissue engineering was enhanced by cell seeding on fibrin scaffold (Ho et al., 2010). Fibrin is a preferred natural and cross-linked structure due to its high water content and low immunogenicity potential (Landers et al., 2002). Fibrin hydrogel facilitates the diffusion of nutrients and metabolites (Landers et al., 2002). Presence of factor XIII in the fibrin enhances mesenchymal stem cell (MSC) proliferation and migration (Catelas et al., 2006). Researchers (Jung et al., 2010) assessed the process of chondrogenesis by applying human adipose derived stem cells (hASCs) in the fibrin scaffold. 

TGF-β members are selective chondrogenic inducers in tissue engineering studies, while, they trigger post-differentiation hypertrophy (Valcourt et al., 2002). If the differentiation process by TGF-β continues for a long time, chondrocyte hypertrophy would be inevitable (Mueller et al., 2010). *In vitro* and *in vivo* conditions such as mechanical loading and inflammation promote hypertrophy of chondrocytes. TGF-β is not able to control these conditions (Davidson et al., 2005). Inflammatory cytokines could influence the TGF-β signaling and amplify hypertrophic phenotype of chondrocytes (Blom et al., 2009). Finding new inducers like the herbal compounds that have anti-inflammatory properties is needed to solve the issue.

Pomegranate (*Punica granatum*) is native to Iran and some regions of India (Chandra et al., 2010). Pomegranate fruit extract (PFE) is rich in nutrients and contains phytochemicals like flavonoids (Miguel et al., 2010). The pomegranate fruit consists of 80% juice and 20% seed (Akpinar-Bayizit et al., 2012). The oily phase is extracted from the seeds containing polyphenolic compounds with strong antioxidant properties. PFE consumption can inhibit the proliferation and invasion of tumor cells (Longtin, 2003; Toi et al., 2003). PFE reduced destructive changes of the chondrocytes and proteoglycans in an osteoarthritic mouse model (Khalifé and Zafarullah, 2011). A research revealed the chondrogenic effect of PFE on the mesenchymal cells isolated from mouse limb bud (Monsefi et al., 2012). PFE promoted cell viability and increased the number and diameter of cartilage nodules (Monsefi et al., 2012). Stimulation of COLII synthesis following culture of hASCs by PFE treated medium was investigated (Katani et al., 2018). Pomegranate concentrated powder (PCP) was consumed to reduce inflammation and increase the articular chondrocytes proliferation (Kang et al., 2015).

In this study, PFE was used to induce hASCs on the fibrin scaffold. The constructs were transplanted in rat knee articular defects and repaired tissues were examined after 8 weeks.

## Materials and Methods

**The study design**

Three groups were assessed both *in vitro* and *in vivo*. The labeled hASCs were seeded on the fibrin scaffold at 5×10^5 ^cells per scaffold. The TGF-β and PFE groups were treated with 10 ng/ml TGF-β and 10 µg/ml PFE, respectively. A control group, which only received chondrogenic medium, was assessed as well. Three groups were compared after 14 days. The adult male rats were divided based on *in-vitro* samples. Scaffolds were transplanted in the rat trochlear groove defects. 

The chemical components applied in this study were from Sigma- Aldrich CO (St. Louis, MO, USA). 

**Preparing PFE**

 Three kilos of pomegranate were purchased from Najaf Abad, Isfahan, Iran. The pomegranate juicy seeds were separated, dried completely, and powdered. The powder was soaked in 70% ethanol, placed on a shaker for 4 hrs and the hydroalcoholic extract passed through a Buchner funnel. The process was repeated 3 times and the extract was concentrated in a 60-rpm rotary machine at 45℃. Finally, the extract was freeze-dried. Upon need, the extract was diluted and sterilized using a 0.22 μm filter (Hajhashemi et al., 2010). 

**Preparation of fibrin**

Fresh Frozen Plasma (FFP) and cryoprecipitate (Cryo) bags were provided from the blood transfusion center of Isfahan and melted completely in a water bath at 37℃. The FFP was then mixed with calcium gluconate (Sina Daru Lab- Iran) at 16/10 w/w in a 50 ml falcon tube and then incubated at 37℃ for 60–90 min. After centrifugation at 580 g for 10 min, the upper clear liquid containing thrombin was collected (Yang, Wu, Shih, Chen, & Lin, 2008). Cryo was used as a source of fibrinogen (15 mg/ml). 

**ASCs isolation and culture**

Subcutaneous fat tissues (~120 g) were obtained from three young cases of abdominoplasty surgery. Tissues were rinsed with phosphate buffered salin (PBS) containing 100 mg/ml streptomycin and 100 IU/ml penicillin (Gibco, Germany), and minced with a sterile knife. Pieces were digested by 0.075% collagenase 1 solution (Gibco, Germany) at 37℃ for 1 hr. The homogenate was centrifuged at 400 g for 10 min. The cellular pellet was cultured at 10^3^ to 10^4^ cells/ml in T-75 flasks which were placed in an incubator with 5% CO_2_ at 37°C. Culture mediums including Dulbecco’s modified Eagle medium (DMEM), 10% fetal bovine serum (FBS), and 1% antibiotics were refreshed every 2-3 days. When 80% of the flask bottom was occupied by hASCs, they were subcultured to provide the required cell count. The cluster of differentiation (CD) markers CD 14, CD 45, and CD 44 of the hASCs were identified (Teimourinejad et al., 2020).

**Labeling of hASCs with CM-DiI**

Cell Tracker^TM^ CM-DiI vial (chloromethyl benzamidodialkylcarbocanin, 1 mg of solid, C-7001, Thermofisher, USA), was stored at -20°C. The stock solution was prepared according to the manufacturer’s instructions. Immediately before labeling, a working solution was prepared in PBS (4 µm). One milliliter of the solution was used for the labeling of 1×10^6^ cells. After the last passage, the cells were first incubated in labeling solution for 5 min at 37°C and next, for 15 min at 4°C. The hASCs were rinsed to remove the excess dye (Xue et al., 2010).

**Chondrogenic induction**

hASCs obtained in passage 3 were counted, labeled, and suspended in the Cryo solution at 1×10^6^ cells/ml. Then, 500 µl of cell/Cryo suspension and an equal volume of the provided thrombin were mixed in a well of the 24 well culture plate. The scaffolds were first incubated at 37°C for 10 min and next, divided into three groups consisting of Control, PFE and TGF-β in a random manner. Chondrogenic medium, containing high glucose DMEM supplemented with 1% antibiotics, 100 nM dexamethasone (Sigma-Aldrich, St Louis, Mo.), 50 μg/ml ascorbate-2-phosphate, 6.25 μg/ml insulin, 6.25 μg/ml transferrin, 6.25 ng/ml selenious acid, 40 μg/ml proline, 100 μg/ml pyruvate, 1.25 mg/ml bovine serum albumin [BSA] and 5.35 mg/ml linoleic acid) was added to each control sample of 1 ml well. 10 µg/ml PFE was diluted in the chondrogenic medium of the PFE group shortly before use (Monsefi et al., 2012). 10 ng/ml TGF-β3 was added to the chondrogenic medium of the TGF-β group. The culture media were refreshed every 2–3 days for 14 days (Sekiya, Vuoristo, Larson, & Prockop, 2002).

**Cell viability (MTT assay)**

 The medium was completely aspirated and constructs were incubated with MTT (3-(4, 5-dimethyl) thiazol-2-yl-2, 5-dimethyl tetrazolium bromide) reagent, 5 mg/ml at 37°C for 4 hr. The constructs were treated with 400 μl of dimethyl sulfoxide (DMSO) for 15 min at room temperature. The absorbance was measured at OD=590 nm. 

**Assessment of chondrogenic differentiation by real-time PCR**

*COL2A1*, *Aggrecan (ACAN), *and hypertrophic marker *COL10A1* expressions were assessed. Extraction of the total RNA from the constructs was accomplished according to the manufacturer’s instructions (Yekta Tajhiz Kit, Tehran, Iran). The quantity and quality of the isolated RNA were determined by Nanodrop 2000 (Thermoscientific). The complementary DNA was synthesized using the oligo dT primers and Biofact 2X One-step RT PCR MasterMix kit (Biofact, Korea). The primers were used according to a previous research (Table 1) (Hashemibeni et al., 2018). The PCR protocol was established using Biofact 2X Real-time PCR Master Mix (High ROX) containing SYBR Green (Biofact, Korea) and the StepOnePlus™ Real-time PCR detection System (Applied Biosystems). The PCR amplification conditions were 15 min at 95°C followed by 40 cycles of denaturation step at 95°C for 20 sec, annealing, and extension for 1 min at 60°C. These experiments were run in triplicate independently, and repeated at least for three times. The *GAPDH* was applied as an endogenous control. The expression level of each target gene was calculated as 2^−ΔΔCt^.

**Table 1:** List of primers applied in RT-qPCR

**Gene**	**Forward Primer (5′ to** **3′)**	**Reverse Primer (5′ to** **3′)**
***COL2A1***	CTGGTGATGATGGTGAAG	CCTGGATAACCTCTGTGA
***ACAN***	GTGGGACTGAAGTTCTTG	GTTGTCATGGTCTGAAGTT
***COL10A1***	AGAATCCATCTGAGAATATGC	CCTCTTACTGCTATACCTTTA
***GAPDH***	TTGAGGTCAATGAAGGGGTC	GAAGGTGAAGGTCGGAGTCA

**Histological assessment of** ***in vitro*** **samples**

The constructs were harvested on day 14 of chondrogenic induction and fixed in 10% formalin for 24 hr. After paraffin embedding, sections of 5 µm thickness were made. General morphology by Haematoxylin and Eosin (H&E) staining and proteoglycan contents by Safranin-O/Fast Green protocol were assessed. Safranin-O protocol was conducted as follows: the sections were first stained with Wiegert’s iron hematoxylin solution for 10 min, next, stained with fast green for 5 min, then, rinsed in 1% acetic acid, and finally, stained with 0.1% aqueous Safranin-O for 10 min.

**Experimental protocol**

The animal protocols were conducted according to the instructions of animal experimentation at Isfahan University. Eighteen adult male rats of 250-300 g (Royan Institute, Isfahan, Iran) were randomly divided into 3 equal groups. The groups were named as *in vitro* to transplant the corresponding constructs at their knee joints. The animals were anesthetized with ketamine 87 mg/kg and xylazine 13 mg/kg and their knees were shaved and disinfected. A lateral longitudinal Para patellar skin incision was made to expose the joint, and the knee capsule was incised. The patella was gently pushed medially and a defect of 1.5 mm width was made in the trochlear region by a biopsy punch (Dahlin et al., 2014). The defect was irrigated by isotonic saline. The construct was placed into the defect. The patella was relocated and the wound was sutured layer by layer. Also, 14 mg/kg/day cyclosporine (Zahravi Pharm CO, Tabriz, Iran) was injected subcutaneously to prevent transplant rejection. During all processes, the rats remained active in their cages. 

**Macroscopic examination of the lesion** **area**

 Eight weeks after the surgery, the animals were anesthetized and sacrificed. The distal femur was dissected and inspected *in situ*. The photographs were taken by a digital camera (Canon, Japan), and the best of them were chosen in terms of quality and blindly scored by three observers through the scoring system (Goebel et al., 2012) . This scoring system evaluates five parameters in repair tissue (presence of blood vessels, color, surface of repaired tissue, filling of the defect and degeneration of adjacent cartilage). Grades ranges from 0 (excellent repair) to 20 (without repair tissue).

**Histological examination and immunofluorescence staining**

The distal femur was fixed in paraformaldehyde 4% and decalcified in 5% formic acid. After dehydrating and embedding in paraffin, sagittal sections of 5 μm thickness were prepared for hematoxylin and eosin (H&E), Safranin-O, and toluidine blue staining. As to toluidine blue staining, sections were stained in toluidine blue solution for 5 min and the excess stain was removed using filter paper. For Safranin-O staining, sections were stained with a 0.1% Safranin-O solution. Slides were observed through a light microscope (Olympus, Japan).

The immunofluorescence method involving cutting 4 μm sections and antigen retrieval by 0.1% pepsin in PBS for 15 min at 37°C, followed by treating with 3% BSA, was adopted. The sections were incubated with 10 μg/ml goat anti-human COLII antibodies (ab24128; Abcam) at 4°C overnight, followed by rinsing and incubation with 10 μg/ml FITC rabbit anti-goat secondary antibody (31509; Invitrogen) for 1 hr at room temperature. The DNA was labeled with DAPI (4, 6-diamidino-2- phenyl indole hydrochloride) solution. Negative controls without primary antibodies were stained in parallel. Slides were imaged through an Axiovert 200M fluorescence microscope (Carl Zeiss Light Microscopy, Germany).

**Statistical analysis**

The data are expressed as the mean± SD and were analyzed through the One-way ANOVA, kruskal-wallis and tukey post hoc test using SPSS s/w. (p<0.05) were significant. 

## Results

**Figure 1:**
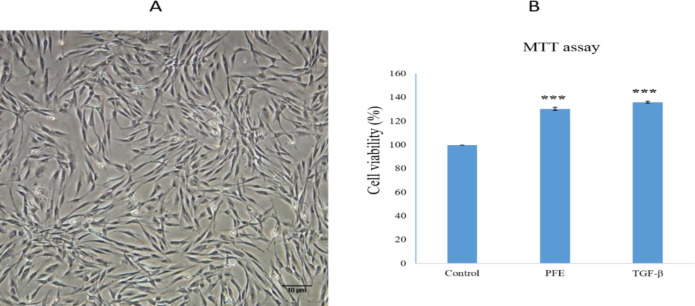
Invert microscopy presentation of monolayer hASCs at passage 3; A; magnification 200 . B; MTT assay results on day 14 of induction. The symbol *** indicates a significant difference from the control group (p<0.001).

hASCs were assessed in the 3rd passage. Cells appeared in spindle shape and they were uniformly spread (Figure 1A). No sign of contamination by microorganisms was observed before harvesting the hASCs. Animals were under observance during first days after transplantation. No major temperature or mobility alteration was observed. 

**MTT assay**

The MTT assay of hASCs induction was run on day 14 for the metabolic activity assessment. The PFE and TGF-β group cell viability was considerably different from the control group (p<0.001). There was no significant difference between PFE and TGF-β groups (Figure 1B). 

**Real-time PCR**

**Figure 2:**
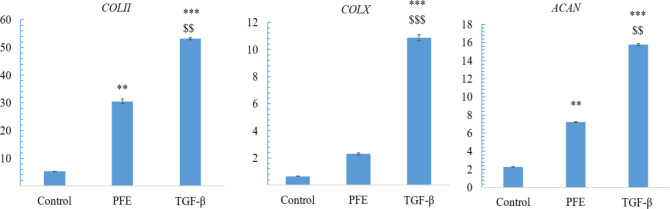
Real-time PCR results of* COL II, ACAN *and* COLX *genes: the symbol * indicates a significant difference from the control (p<0.05), the symbol $ indicates a significant difference from the PFE (p<0.05).

Cartilage specific genes (*COLII* and *ACAN*) were assessed. A significantly higher expression of these genes in the PFE and TGF-β groups than the control (p<0.001) was evident. The *COLII* level and *ACAN* expression in the TGF-β group was significantly different from the PFE group (p<0.01). The *COLΧ* expression, as a hypertrophy marker in the TGF-β and PFE groups was significantly higher than that of the control (p<0.001). The *COLΧ* expression in the TGF-β group was different from the PFE group (p<0.001) (Figure 2).

**Histology results** ***in vitro***

**Figure 3:**
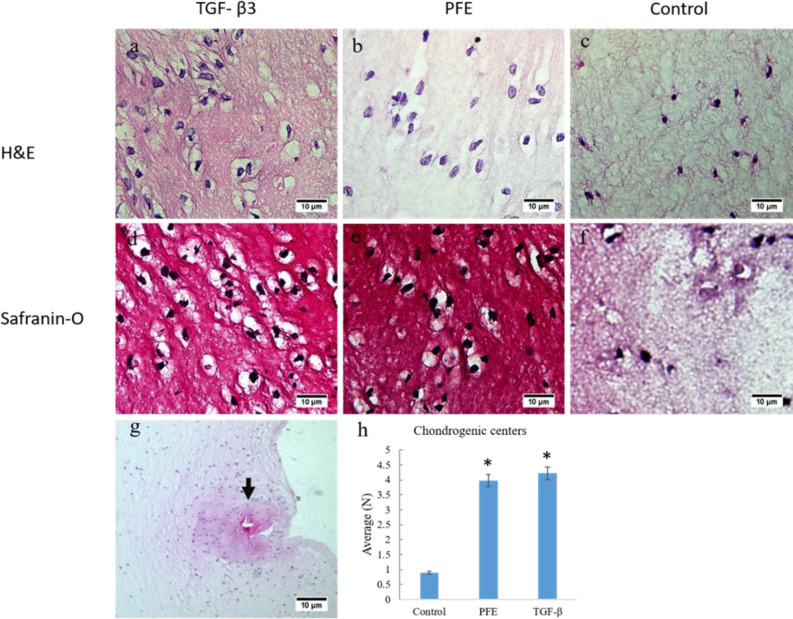
*In-vitro* histologic assessment of the constructs on day 14 of differentiation. Red stain intensity in Safranin-O is evidenced by glycosaminoglycan volume secreted by chondrogenic cells. Remarkable hypertrophy is observed in some of the TGF-β3 induced cells (Magnification 40х). A chondrogenic center (Arrow) at low magnification (10х) is shown in Figure 4g. The chart represents the chondrogenic centers average count. ***shows significant difference from the control (p<0.001).

Histological slides exhibiting chondrocytes and matrix were prepared (Figure 3 a-c), the chondrocytes were less in the control samples (Figure 3c). The number of chondrogenic centers was counted (Figure 3h), and their average was different significantly between the PFE and control groups (p<0.05) Also, the average of chondrogenic centers did not show a significant difference between the TGF-β and PFE groups (p>0.05). The intensity of safranin-O staining, indicating of cartilage matrix production, was prominent in the TGF-β3 (Figure 3d) and PFE (Figure 3e) groups. 

**Gross assessment**

**Figure 4:**
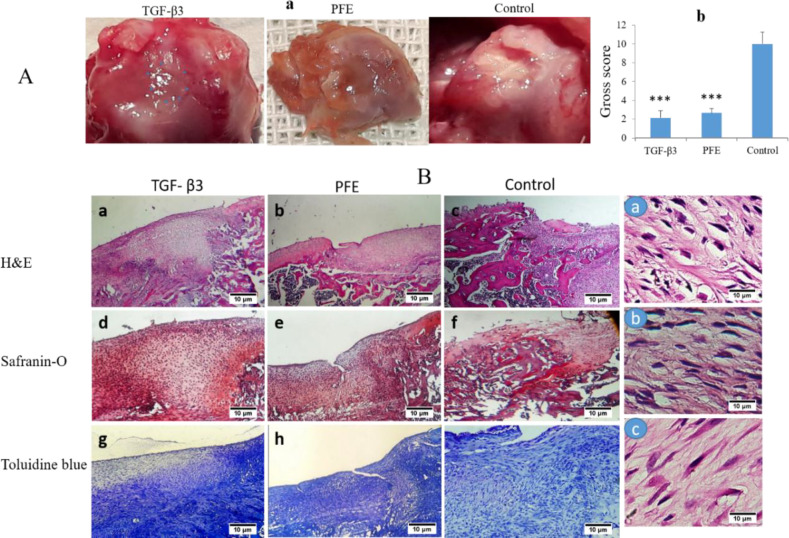
*In-vivo* assessment of the samples at the end of week 8 post transplantation: macroscopic, images (Figure 4A/a) shows defect filling with a tissue the same color as the surrounding cartilage in the PFE and TGF-β3 groups. White fibrous tissue and obvious degeneration were observed in the control sample. The chart in (Figure 4A/b) shows significant difference between the two groups and the control (p<0.001). Histological image shows cartilaginous tissue within the defects of the TGF- β3 and PFE groups (Figure 4B/left). Figure 4B/right shows magnified sections of the TGF- β3 (With hypertrophic cells) (a), PFE (b) and control (c) samples (40x).

Defect repairing evaluation showed no evidence of rejection or severe inflammation. Images were selected in terms of quality and viewed by three independent evaluators. The parameters were compared among the groups and the average score was calculated. The filled defects without irregularity were observed in the TGF-β samples and coloration of the repaired tissues was such as the adjacent cartilage (Figure 4A). Both TGF-β3 and PFE specimens had significant differences from the control (p<0.05). There were no significant differences between the TGF- β and PFE groups in the gross assessment of samples (Figure 4A). 

**Histochemical findings (** ***in vivo*** **)**

General appearance was assessed through H&E staining. Integrity and connectivity of the repaired tissues with surrounding native cartilage, and cell shape and density were assessed (Figure 4B). Slides prepared from the TGF- β (a, d, and j), and PFE (b, e, and h) samples exhibited an obvious connection with the neighboring cartilage and bone. Chondrocytes and in some cases, long cells were observed, (Figure 4B). a few of chondrocyte-like cells were seen in the control samples (c, f, and i). Consistent with gross morphology and general histological view, the evaluation of toluidine blue and Safranin-O staining revealed more severe staining in the TGF-β and PFE groups, indicative of high extracellular matrix secretion in the new-formed tissues (Figure 4B).

**Immunofluorescence assessment of the neo-formed tissues**

**Figure 5:**
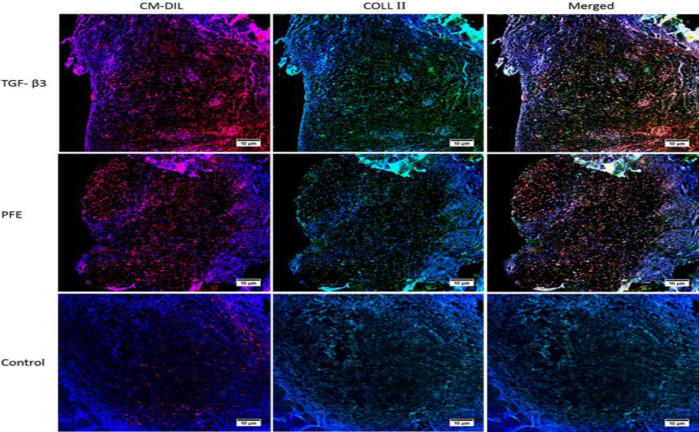
Immunofluorescence study of repaired tissue 8 weeks after the transplantation. The CM-DiI-labeled human cells identified in red. The green color is indicative of human *COLII* expression that is considerable throughout TGF-β_3_ and PFE samples. Few CM-DiI labeled cells are observed in the control group image. The host cells were not labeled.

Transplanted host cells were identified due to the red labeling by CM-DiI. The positivity of COLII protein was determined as Green. The immunofluorescence assessment revealed human COLII protein produced by differentiated hASCs. The COLII production in the tissues evolved from the control transplants was not noticeable and less labeled cells were observed (Figure 5).

## Discussion

The results of the MTT assay indicated a significant difference between the PFE and control samples (p0.001) and there was no significant difference between the PFE and TGF-β3 (p0.5) on day 14 of differentiation (Figure 1B).

The expression of cartilage-specific genes in the PFE group was at a high level (Figure 2). The expression of hypertrophic marker *COLΧ* in the PFE group was significantly lower than that of the TGF-β3 group (p0.001). A significant increase in *COLIІ* expression by PFE was revealed in this study.

The comparison of the histochemistry results of *in vitro* samples (Figure 3h) exhibited a significant difference between the PFE and control samples (p<0.001). There was no significant difference in the chondrogenic center count between the PFE and TGF-β3 samples (p>0.05). The Safranin-O staining results were compared among three groups in qualitative sense. Red intensity exhibited higher levels of glycosaminoglycans in the PFE and TGF-β3 groups (Figure 3d-f).

A macroscopic reverse scaling table was applied to compare the parameters among the groups. Grades obtained from the transplanting areas of the distal femur (Figure 4A) revealed a significant difference between the PFE and control samples (p<0.001). 

Many chondrocytes and dense matrices were seen at the cross-sections of the PFE samples (Figure 4B). The red stain of Safranin-O and the metachromatic reaction of toluidine blue were prominent in the PFE and TGF-β3 sections (Figure 4B), confirming the presence of sulfate and phosphate groups produced in high concentration by differentiated hASCs.

COLII produced by chondrocytes of the constructs was detected through immunofluorescence staining (Figure 5). The CM-DiI-labeled hASCs on fibrin scaffold differentiated by PFE treated medium were positive for COLII protein after transplantation. Actually, chondrocytes differentiated from hASCs proliferated in the process of tissue repair. 

Previous *in-vitro* and *in-vivo* studies (Ahmed et al., 2005; Shukla et al., 2008a, 2008b) also used pomegranate extract to preserve articular chondrocytes. Pretreatment of the human osteoarthritic chondrocytes with PFE significantly reduced interleukin1-β toxicity. Adding PFE to the cultured chondrocytes prevented the degradation of cartilage and reduced proteoglycan release (Ahmed et al., 2005; Shukla et al., 2008b). Oral consumption of PFE by osteoarthritic mice model significantly reduced inflammatory cytokines, and interleukin 6 (IL-6) and arthritis severity (Shukla et al., 2008a). These researches revealed the effectiveness of the bioavailable metabolites of PFE in nitric oxide production and PGE2 in the chondrocytes. 

Researchers (Cerdá et al., 2006; Patel et al., 2008) have focused on the contribution of PFE to chondrogenesis. The findings of the present study corresponded with the previous one which assessed the *in-vitro* differentiation of hASCs by PFE (Katani et al., 2018). The chondrogenic differentiation of mouse limb bud stem cells under the effect of PFE was assessed (Monsefi et al., 2012) and enhancement of the viability rate of cultured cells and formation of cartilage nodules using PFE were found. The finding of this study corresponded with that of the present study. 

High cell viability in the PFE samples may be due to the rich polyphenol contents of PFE (Cerdá et al., 2006; Patel et al., 2008). Proliferation may be correlated with pre-differentiation of stem cells and chondroblasts mitosis activation.

Chondrocyte hypertrophy is a common consequence of the differentiation by the TGF-β family (Risbud and Sittinger, 2002; Steinert et al., 2007). Hypertrophy of chondrocytes is determined by increasing the cell volume and *COLΧ* expression (Ho et al., 2010; Steinert et al., 2007). In the present study, hypertrophied cells were clearly observed in TGF-β3 tissue samples (Figure 3 and 4). Despite the significantly higher level of *COLΧ* expression in the TGF-β3 samples than that of the PFE samples, there was no considerable difference between the *in-vivo* results of the two treated groups. This discrepancy can be described by the short period of *in vitro* examination. The TGF-β3 is required for all stages of chondrocyte differentiation, and 14 day period is probably not enough to advance chondrocytes towards final differentiation (Mueller et al., 2010). 

In addition to chondrocytes, the elongated cells were observed in the histological sections of *in-vivo* samples. This is probably due to the forces applied on the defect area during rats’ movement and the gel-like consistency of the fibrin scaffold (Figure 4A). 

In general, macroscopic and microscopic assessment of the study proved that pre-differentiation of hASCs under the induction of PFE is effective in post-transplantation repair. Because of its constituents such as polyphenol ingredients, PFE affected cartilage repair. This may be due to the existence of anti-oxidant and anti-inflammatory compounds (Garbacki et al., 2002; Seeram and Nair, 2002). 

The molecular pathways by which the chondrogenic effect of PFE is exerted, are not well known, thus necessitating further investigations. 

In this study, PFE was applied as a chondrogenic inducer of hASCs. The results extend our knowledge of the herbal extract and encourage its consumption for future studies.

## Conflicts of interest

The authors have declared that there is no conflict of interest.
